# Antiproliferative Properties of a Few Auranofin-Related Gold(I) and Silver(I) Complexes in Leukemia Cells and their Interferences with the Ubiquitin Proteasome System

**DOI:** 10.3390/molecules25194454

**Published:** 2020-09-28

**Authors:** Damiano Cirri, Tanja Schirmeister, Ean-Jeong Seo, Thomas Efferth, Lara Massai, Luigi Messori, Nicola Micale

**Affiliations:** 1 Department of Chemistry and Industrial Chemistry (DCCI), University of Pisa, Via Moruzzi 13, 56124 Pisa, Italy; damiano.cirri@unifi.it; 2 Institute of Pharmaceutical and Biomedical Sciences, Johannes Gutenberg University, Staudinger Weg 5, 55128 Mainz, Germany; schirmei@uni-mainz.de; 3 Department of Pharmaceutical Biology, Institute of Pharmaceutical and Biomedical Sciences, Johannes Gutenberg University, Staudinger Weg 5, 55128 Mainz, Germany; seo@uni-mainz.de (E.-J.S.); efferth@uni-mainz.de (T.E.); 4 Department of Chemistry “Ugo Schiff”, University of Florence, Via della Lastruccia 3-13, 50019 Sesto Fiorentino, Italy; lara.massai@unifi.it; 5 Department of Chemical, Biological, Pharmaceutical and Environmental Sciences, University of Messina, Viale Ferdinando Stagno D’Alcontres 31, I-98166 Messina, Italy

**Keywords:** auranofin, metal complexes, proteasome inhibition, leukemia cells, antiproliferative properties

## Abstract

A group of triethylphosphine gold(I) and silver(I) complexes, structurally related to auranofin, were prepared and investigated as potential anticancer drug candidates. The antiproliferative properties of these metal compounds were assessed against two leukemia cell lines, i.e., CCRF-CEM and its multidrug-resistant counterpart, CEM/ADR5000. Interestingly, potent cytotoxic effects were disclosed for both series of compounds against leukemia cells, with IC_50_ values generally falling in the low-micromolar range, the gold derivatives being on the whole more effective than the silver analogues. Some initial structure-function relationships were drawn. Subsequently, the ability of the study compounds to inhibit the three main catalytic activities of the proteasome was investigated. Different patterns of enzyme inhibition emerged for the various metal complexes. Notably, gold compounds were able to inhibit effectively both the trypsin-like and chymotrypsin-like proteasome activities, being less effective toward the caspase-like catalytic activity. In most cases, a significant selectivity of the study compounds toward the proteasome proteolytic activities was detected when compared to other proteases. The implications of the obtained results are discussed.

## 1. Introduction

**Figure 1:**
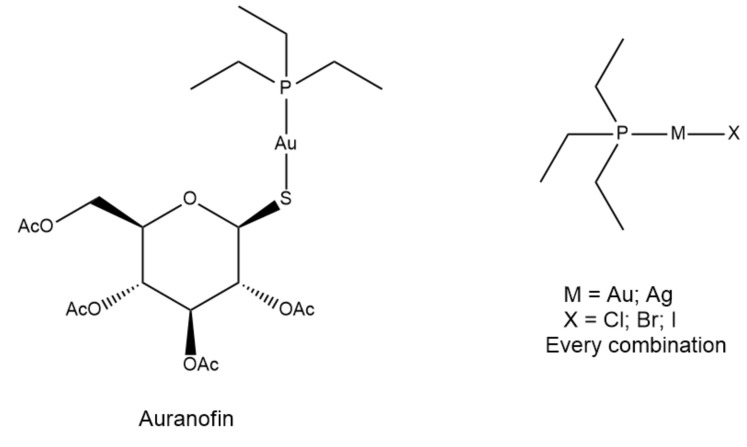

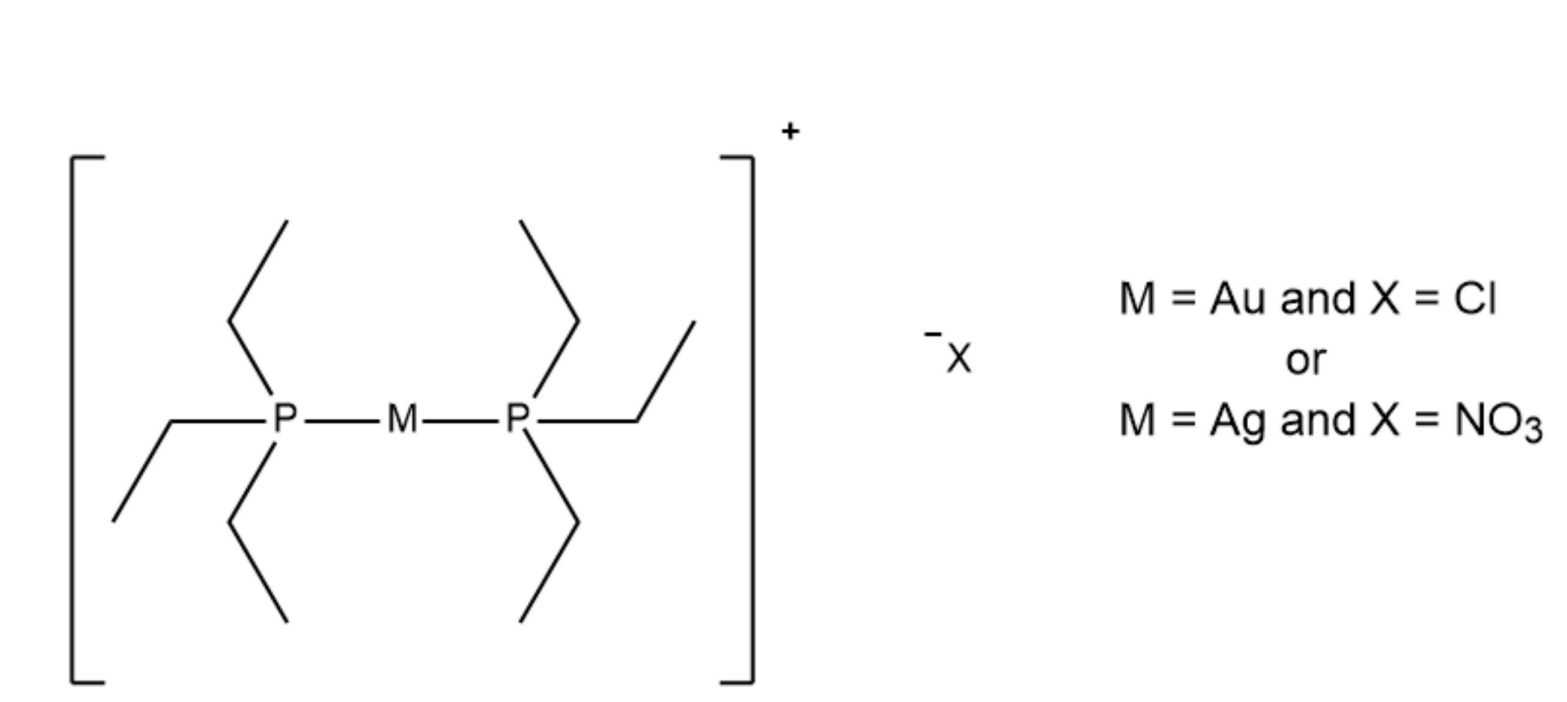
The panel of metal complexes studied in this work: Auranofin (reference compound), Au(I) and Ag(I) monophosphine derivatives of general formula M(PEt_3_)X and diphosphine derivatives of general formula [M(PEt_3_)_2_]X.

Auranofin (AF), an established gold(I) drug approved for the treatment of rheumatoid arthritis, has been reported to manifest relevant antiproliferative actions against a variety of cancer cell lines [1]. These observations fueled a lot of investigations on the anticancer properties of AF and its analogues, and on the underlying molecular mechanisms [2,3,4,5]. In recent years, new knowledge regarding the molecular mechanisms involved in leukemia cells growth has shifted the focus of antileukemic drug development to agents acting on specific molecular targets and pathways that regulate signal transduction, epigenetic alterations, cell death, and cell cycle progression [6,7,8,9]. In this regard, the ubiquitin-proteasome system seems to be a particularly attractive target [10]. The ubiquitin-proteasome system maintains protein homeostasis by controlling the degradation of damaged, abnormally folded, or short-lived regulatory proteins. Consequently, it has the capacity to influence key cellular functions related to progression through the cell cycle, cellular proliferation, cellular differentiation, DNA damage repair, and cell death [11]. Elevated proteasomal activity has been demonstrated in many cancers, particularly in hematological neoplasms, because malignant cells are often heavily dependent on proteasomal function, particularly for the degradation of proteins that hinder proliferation [12]. Thus far, the proteasome has been shown to be a validated therapeutic target in multiple myeloma and lymphoma, and a range of proteasome inhibitors have been, and continue to be, evaluated for clinical efficacy including a few peptide boronates (e.g., bortezomib, delanzomib and ixazomib) [13]. Remarkably, bortezomib and ixazomib, together with the peptide epoxyketone carfilzomib, have been approved by the US Food and Drug Administration (FDA) for multiple myeloma patients who progress after initial treatment. Based on these arguments, we have further explored whether a selected group of metal compounds might contrast the growth of leukemia cells in vitro as well as inhibit potently and selectively the main protease activities of the proteasome. Previous results showed that, in general, metal-based compounds endowed with anticancer activity may behave as strong proteasome inhibitors [14,15,16]. In particular, pertaining to anticancer gold derivatives, we reported that a group of gold(III) complexes and a gold(I) phosphine complex caused profound and differential inhibition of this target, at variance with the reference compound AF, which turned out to be nearly ineffective [17]. These results point out that the proteasome could be a common target that may contribute to mediate the cytotoxic activity of many gold compounds. Moreover, in this case, our cross-reactivity investigations on the above compounds showed a notable selectivity only toward bovine pancreatic α-chymotrypsin, whereas the whole set of gold complexes inhibited the human cysteine proteases cathepsin-B (Cat-B) and cathepsin-L (Cat-L) to a certain extent. Accordingly, we have prepared a series of linear metal complexes structurally related to AF (i.e., halide or diphosphine analogues), containing either gold or silver as the central metal, and investigated their antiproliferative properties in two leukemia cell lines, namely the drug-sensitive CCRF-CEM cells and their multidrug-resistant P-glycoprotein-over-expressing sub-cell line CEM/ADR5000, as well as their ability to inhibit the catalytic activities of the proteasome. The aim of the work was to verify how the partial modification of the reference compound might affect the anticancer profile and the proteasome inhibition patterns. The selectivity of inhibition was further checked by analyzing the effects of the same metal compounds on the above-mentioned protease activities (cross-reactivity analysis). Interestingly, quite favorable pharmacological profiles emerged for some of the study compounds that warrant further evaluation. The investigational panel included auranofin and eight additional silver and gold complexes related to it (Figure 1). The synthesis, characterization and solution behavior of these molecules were previously described [18,19].

## 2. Results and Discussion

### 2.1. In vitro Inhibition of Cancer Cell Growth

First, we analyzed the antiproliferative properties of the panel compounds in vitro against two representative leukemia cell lines, i.e., the drug-sensitive CCRF-CEM and its multidrug-resistant counterpart CEM/ADR5000. The two leukemia cell lines were selected for their studied genotypes and gene expression profiles [20]. Results are shown in Table 1. We observed the following:

The gold-containing compounds (i.e., AF, its halide derivatives and the diphosphine cationic compound; see Table 1) were found to produce the greatest cytotoxic effects with IC_50_ values falling in all cases in the 200–300 nM concentration range. The different halides or the second phosphine ligand are found to scarcely affect the antiproliferative potency. These data suggest that in all cases the pharmacophore is basically the same and should correspond most likely to the [Au(PEt_3_)]^+^ moiety. The two leukemia cell lines were equally sensitive toward the various gold compounds, implying that the molecular processes leading to multidrug resistance do not reduce cells’ sensitivity to these gold compounds. Importantly, this type of complex does not seem to significantly affect the cell viability of healthy cells (IC_50_ > 5 μM) as previously assessed for auranofin, Au(PEt_3_)Cl and Au(PEt_3_)I towards human fibroblast cells (HDF) and human embryonic kidney cells (HEK293) [18].

Generally, the silver derivatives were less effective than the gold counterparts by a factor ~10 (Table 1). Again, the antiproliferative effects of the silver compounds scarcely depended on the nature of the halide ligand. At variance with gold compounds, some degree of cross-resistance with the multidrug-resistant cell line was detected for silver compounds, though relatively low (1.14–3.05). The reported greater cytotoxic potency of gold compounds as compared to silver compounds is in line with previous observations carried out in a panel of ovarian cancer cell lines [21]. For both series of compounds, the obtained cytotoxicity data suggest that the [M(PEt_3_)]^+^ moiety probably represents the “true pharmacophore”. Indeed, the second ligand seems to not significantly affect the antiproliferative properties; on the contrary, the nature of the metal plays a crucial role in modulating the anticancer activity. Afterward, the three main catalytic activities of the proteasome, i.e., chymotrypsin-like (ChT-L), trypsin-like (T-L) and peptidyl-glutamyl peptide-hydrolysing (PGPH; also known as caspase-like C-L) were monitored and their inhibition by panel compounds was determined. The proteolytic activities are exerted by the subunits β5 (Ch-T), β2 (T-L) and β1(PGPH), which are present in the human 20S proteasome. Results are shown in Table 2 in terms of the obtained IC_50_ values (μM) or % of inhibition at 10 μM. Both the gold and silver halide derivatives showed an outstanding inhibition profile; notably, only two out of the three proteolytic activities of the 20S proteasome (i.e., ChT-L and T-L) are affected. We can argue that the inhibition of all three proteolytic activities of the target may result in cytotoxicity also in normal cells, whereas the co-inhibition of the ChT-L activity (i.e., the β5-subunit, the primary tumoral target) with a second catalytic activity of the proteasome is crucial for the achievement of an ideal anticancer activity [22]. These two proteolytic activities were strongly inhibited by the three gold halide derivatives (low-micromolar range). The silver complexes caused an important inhibition of the T-L activity, nearly comparable to the gold analogues, but turned out to be significantly less effective toward the ChT-L activity. The two diphosphine derivatives instead, showed only a modest inhibition of the proteasomal ChT-L activity. Notably, the C-L activity was moderately sensitive to gold halide compounds and insensitive to the silver analogues. These results suggest that the study compounds may produce a relevant inhibition of some of the catalytic activities of the proteasome, but inhibition typically occurs at concentrations about 1 order of magnitude greater than those needed to inhibit leukemia cell growth. Also, these data suggest that the inhibition of the proteasome is not the main and only target for these metal complexes; they hold most likely a multitarget cytotoxic activity, as described in several papers [23,24].

### 2.2. Cross-Reactivity Analysis

The selectivity of the inhibitory effects of the study compounds towards the proteasome was also documented by performing cross-reactivity assessments. Selectivity is commonly proved by establishing whether the compounds that are effective inhibitors of the proteasome activities are also able to inhibit some other proteases involved in physiological processes. Specifically, to prove such selectivity, we considered the following proteases: Bovine pancreatic α-chymotrypsin, human cathepsin-B (Cat-B) and human cathepsin-L (Cat-L). Notably, we observed that all study compounds were on the whole ineffective in contrasting these three enzyme activities, with an exception made for three gold halide derivatives that acted as moderate inhibitors for human Cat-B (~30%), and for the two diphosphine derivatives [Au(PEt_3_)_2_]Cl and [Ag(PEt_3_)_2_]NO_3_, which slightly inhibited bovine pancreatic α-chymotrypsin (~20%) and human Cat-L (~20%), respectively (Table 3).

## 3. Materials and Methods

### 3.1. Cell Culture 

Drug-sensitive CCRF-CEM and multidrug-resistant (MDR) P-glycoprotein (P-gp)-over-expressing CEM/ADR5000 leukemic cells were kindly provided by Prof. Axel Sauerbrey (Department of Pediatrics, University of Jena, Germany). Cells were cultured in RPMA1640 medium including 10% fetal bovine serum (FBS) and 1% penicillin (1000 U/mL)/streptomycin (100 µg/mL) (Life Technologies, Darmstadt, Germany). Doxorubicin (5000 ng/mL) was added to maintain overexpression of P-gp (MDR1 and ABCB1) in CEM/ADR5000 cells [26]. Doxorubicin was obtained from the University Pharmacy of the University Medical Center Mainz. CEM/ADR5000 cells display a more than 1000-fold resistance to doxorubicin [27] and are therefore a suitable model to identify novel drugs without or very low cross-resistance.

### 3.2. Cytotoxicity Assay 

The cytotoxic effects of the compounds solved in DMSO (Sigma-Aldrich, Taufkirchen, Germany) were tested by the resazurin assay [28]. This assay is based on reduction of the indicator dye, resazurin, to the highly fluorescent resorufin by viable cells. Cells lethally damaged by cytotoxic compounds rapidly lose the enzymatic capability to reduce non-fluorescent resazurin to the highly fluorescent resorufin. As the reductive potential happens in a dose-dependent manner, this chemical reaction can be exploited to quantitatively determine the cytotoxic potential of investigational compounds and to measure dose-response relationships. With increasing concentrations of a cytotoxic drug, the fluorescence signal fades out. Aliquots of 10,000 cells/100 µL cells were seeded into 96-well plates (Thermo-Scientific, Dreieich, Germany) and immediately treated with various concentrations of each compound. After 72 h incubation at 37 °C, 20 µL resazurin 0.01% *w/v* solution (Invitrogen, Dreieich, Germany) were added to each well, and the plates were maintained at 37 °C for 4 h. Fluorescence was measuring by an Infinite M2000 Proplate reader (Tecan, Crailsheim, Germany) with an excitation wavelength of 544 nm and an emission wavelength of 590 nm. Each experiment was done at least three times with six replicates each. The viability was analyzed in comparison with untreated cells. The 50% inhibitory concentration (IC_50_) is a generally acknowledged parameter to quantify the capability of a test substance to inhibit a biological function of interest. It is a well-established measure to determine the cytotoxic potential of anticancer drugs. The IC_50_ values were calculated from a calibration curve by linear regression using Microsoft Excel [29,30,31].

### 3.3. Enzymatic Assays

20S proteasome, isolated and purified from human erythrocytes, was obtained from a commercial source (Biomol GmbH, Hamburg, Germany), as well as the peptidic substrates (Bachem, Bubendorf, Switzerland) Suc-Leu-Leu-Val-Tyr-AMC, Boc-Leu-Arg-Arg-AMC and Cbz-Leu-Leu-Glu-AMC for ChT-L, T-L and PGPH (C-L) activity of the enzyme, respectively. The three distinct proteolytic activities of the 20S proteasome were measured by monitoring the hydrolysis of the substrates and detecting the fluorescence of the common product released, i.e., 7-amino-4-methyl coumarin (7-AMC), by means of an Infinite 200 PRO microplate reader (Tecan, Männedorf, Switzerland) at 30 °C with a 380-nm excitation filter and a 460-nm emission filter. All gold and silver complexes were tested against the three proteolytic activities of the 20S proteasome and against Cat-B, Cat-L and bovine pancreatic α-chymotrypsin at 10 µM concentrations over a period of 10 min using an equivalent amount of DMSO as a negative control. IC_50_ values were determined after 10 min without pre-incubation of enzyme and inhibitor prior to substrate addition using different inhibitor concentrations from 0.25 µM to 20 µM, ranging from zero to full enzyme inhibition. The IC_50_ values were calculated with the program GraFit^®^ using the two-parameter equation. More detailed experimental protocols for the assays against each proteolytic activity of the 20S proteasome, as well as against bovine pancreatic α-chymotrypsin, are already reported elsewhere (see also Supplementary Materials) [32]. Cat-B and Cat-L were purchased from Calbiochem, Darmstadt, Germany, and the related assays were performed as previously described [33]. The employed fluorogenic substrate for both cysteine proteases was Cbz-Phe-Arg-AMC (80 µM for Cat-B; 5 µM for Cat-L).

### 3.4. Metal Complexes Preparation 

Auranofin and Au(PEt_3_)Cl were purchased respectively from Enzo Life Sciences and Sigma-Aldrich and used without further purifications. The remaining complexes were prepared in our laboratory following already reported procedures [18,19]. All solvents and reagents used during metal complexes preparation were purchased from Sigma-Aldrich (Milan, Italy) and used without further purifications.

## 4. Conclusions

Here, we have evaluated comparatively a group of gold(I) and silver(I) compounds strictly related to auranofin structure, for their ability to inhibit the growth of two distinct leukemia cell lines. We found that all eight metal compounds of the investigational panel are appreciably cytotoxic with IC_50_ values falling in the low-micromolar, and even sub-micromolar range. Apparently, in the series of gold compounds of general formula Au(PEt_3_)X, the nature of the screened X ligand does not affect appreciably the cytotoxic potency; yet, replacement of gold(I) with silver(I) involves a significant attenuation of the cytotoxic properties (by a factor ~10).

Afterward, the study compounds were explored for their ability to inhibit the main catalytic activities of the proteasome, a putative biomolecular target for cytotoxic gold compounds. Specifically, the T-L, ChT-L and C-L activities were monitored; we found that gold compounds behave as strong inhibitors of T-L and ChT-L activities with IC_50_ values in the low-micromolar range. Moderate/low inhibitory effects were instead found for the C-L activity. It must be noted that in the case of gold compounds, IC_50_ values for proteasome inhibition are greater than IC_50_ values for leukemia cells growth inhibition, suggesting that other molecular mechanisms must be operative to induce leukemia cells death. A similar trend with somewhat weaker inhibition effects was observed for the silver analogues. At variance, AF was found not to significantly inhibit the three catalytic activities of the proteasome. Also, we could establish that the effects observed on the proteolytic activities of the proteasome are quite selective; indeed, the same compounds were not able to inhibit some other reference catalytic activities. Overall, these results point out that gold complexes, of general formula Au(PEt_3_)X, possess potent antiproliferative properties toward two representative leukemia cell lines (i.e., CCRF-CEM and CEM/ADR5000 cell lines) being able to overcome multidrug resistance. In addition, we have shown that these metal-based drugs are able to strongly inhibit two of the main enzymatic activities of the proteasome and this effect might contribute to their overall cytotoxic action. Also, some useful structure activity relationships could be derived from comparative analysis of the obtained results in two homologous series of metal complexes. Overall, these interesting results warrant a further and more extensive evaluation of the most promising study compounds.

## Figures and Tables

**Table 1:** Inhibitory effects (IC_50_ values ± SD) of the investigated compounds on cell growth of drug-sensitive CCRF-CEM and of multidrug-resistant CEM/ADR5000 cell lines.

Compound	CCRF-CEM IC_50_ [µM]	CEM/ADR5000 IC_50_ [µM]	Degree of Resistance ^[a]^	n
auranofin	0.22 ± 0.08	0.28 ± 0.06	1.27	3
Au(PEt_3_)Cl	0.35 ± 0.03	0.26 ± 0.04	0.74	3
Au(PEt_3_)Br	0.28 ± 0.06	0.27 ± 0.05	0.96	3
Au(PEt_3_)I	0.32 ± 0.07	0.28 ± 0.08	0.87	3
[Au(PEt_3_)_2_]Cl	0.19 ± 0.06	0.23 ± 0.09	1.21	3
Ag(PEt_3_)Cl	1.36 ± 0.25	2.64 ± 0.48	1.94	3
Ag(PEt_3_)Br	1.50 ± 0.10	3.89 ± 0.70	2.59	3
Ag(PEt_3_)I	1.28 ± 0.12	1.46 ± 0.15	1.14	3
[Ag(PEt_3_)_2_]NO_3_	1.39 ± 0.04	4.24 ± 0.05	3.05	3

[a] The degree of resistance was determined as the ratio of IC_50_ value of multidrug-resistant CEM/ADR5000 cells divided by the IC_50_ value of drug-sensitive CCRF-CEM cells; *n* = number of independent experiments with six replicates each.

**Table 2:** Inhibition (IC_50_ values or % of inhibition at 10 µM) of the chymotrypsin-like (ChT-L), trypsin-like (T-L) and peptidyl-glutamyl peptide-hydrolyzing (PGPH or caspase-like C-L) activities of human 20S proteasome by panel compounds. Inhibition includes standard deviation from three independent experiments, each performed in duplicate.

[IC_50_ Value (µM) or Inhibition (%) at 10 µM]			
Compound	ChT-L	T-L	PGPH (C-L)
auranofin	n.i. ^a^	n.i.	n.i.
Au(PEt_3_)Cl	2.6 ± 0.3 µM	1.25 ± 0.05 µM	>10 µM
Au(PEt_3_)Br	1.4 ± 0.8 µM	1.26 ± 0.12 µM	>10 µM
Au(PEt_3_)I	6.1 ± 0.4 µM	5.80 ± 0.30 µM	>10 µM
[Au(PEt_3_)_2_]Cl	21 ± 1.1 %	n.i.	n.i.
Ag(PEt_3_)Cl	28 ± 0.5 %	2.20 ± 1.30 µM	n.i.
Ag(PEt_3_)Br	22 ± 0.4 %	2.65 ± 0.15 µM	n.i.
Ag(PEt_3_)I	25 ± 0.2 %	2.27 ± 0.78 µM	n.i.
[Ag(PEt_3_)_2_]NO_3_	38 ± 5.0 %	n.i.	n.i.

[a] n.i. = no inhibition. IC_50_ values for the reference compound bortezomib: ChT-L: 38 ± 5.9 nM (ref. [25] 7 nM); T-L: 2206 ± 246 nM (ref. [25] 4200 nM); PGPH (C-L): 84 nM (ref. [25] 74 nM). IC_50_ values in ref. [25] were determined after 1 h; the IC_50_ values for bortezomib in the present study were determined immediately after substrate addition, and are—due to the covalent, time-dependent inhibition by bortezomib—higher for the ChT-L and PGPH (C-L) activities, which are more susceptible to bortezomib.

**Table 3:** Screening at 10 µM of the panel compounds for the inhibition of bovine pancreatic α-chymotrypsin, human Cat-B and Cat-L. Inhibition includes standard deviation from three independent experiments, each performed in duplicate.

Inhibition (%) at 10 µM			
Compound	Bovine Pancreatic α-Chymotrypsin	Cat-B	Cat-L
auranofin	n.i. ^a^	n.i.	n.i.
Au(PEt_3_)Cl	n.i.	29 ± 0.2%	n.i.
Au(PEt_3_)Br	n.i.	31 ± 2.2%	n.i.
Au(PEt_3_)I	n.i.	29 ± 1.7%	n.i.
[Au(PEt_3_)_2_]Cl	20 ± 0.2%	n.i.	n.i.
Ag(PEt_3_)Cl	n.i.	n.i.	n.i.
Ag(PEt_3_)Br	n.i.	n.i.	n.i.
Ag(PEt_3_)I	n.i.	n.i.	n.i.
[Ag(PEt_3_)_2_]NO_3_	n.i.	n.i.	20 ± 0.4%

[a] n.i. = no inhibition.

## Supplementary Materials

The detailed experimental protocols for the enzymatic assays are available online.
