# Supplementary material for: Antiproliferative Properties of a Few Auranofin-Related Gold(I) and Silver(I) Complexes in Leukemia Cells and their Interferences with the Ubiquitin Proteasome System
Source: Molecules. 2020 Sep 28;25(19):4454. doi: 10.3390/molecules25194454 (PMC7582876; doi:10.3390/molecules25194454)
Supplement: Supplementary file 1 [file molecules-25-04454-s001.pdf]

Supporting Information

Antiproliferative properties of a few structure-related gold(I) and silver(I) complexes in leukemia cells and their interferences with the ubiquitin proteasome system

Damiano Cirri [1], Tanja Schirmeister [2], Ean-Jeong Seo [3], Thomas Efferth [3], Lara Massai [4], Luigi Messori [4,*] and Nicola Micale [5,*]

1 Department of Chemistry and Industrial Chemistry (DCCI), University of Pisa, Via Moruzzi 13, 56124 Pisa (Italy); damiano.cirri@unifi.it (D.C.)
2 Institute of Pharmaceutical and Biomedical Sciences, Johannes Gutenberg University, Staudinger Weg 5, 55128 Mainz (Germany); schirmei@uni-mainz.de (T.S.)
3 Department of Pharmaceutical Biology, Institute of Pharmaceutical and Biomedical Sciences, Johannes Gutenberg University, Staudinger Weg 5, 55128 Mainz (Germany); seo@uni-mainz.de (E.-J.S.), efferth@uni-mainz.de (T.E.)
4 Department of Chemistry "Ugo Schiff", University of Florence, Via della Lastruccia 3-13, 50019 Sesto Fiorentino (Italy); lara.massai@unifi.it (LA.M.), luigi.messori@unifi.it (LU.M.)
5 Department of Chemical, Biological, Pharmaceutical and Environmental Sciences, University of Messina, Viale Ferdinando Stagno D'Alcontres 31, I-98166 Messina (Italy); nmicale@unime.it (N.M.)
* Correspondence: luigi.messori@unifi.it (LU.M.), nmicale@unime.it (N.M.)

Inhibition assay for the Chymotryptic Activity of the 20S Proteasome

Human 20S proteasome was incubated at 30 °C at a final concentration of 0.004 mg·mL^{-1} with test compound present at 10 μM (screening assay) or at variable concentrations (continuous assay). The reaction buffer consisted of 50 mM Tris pH 7.5, 10 mM NaCl, 25 mM KCl, 1 mM $MgCl_2$, 0.03% SDS and 5% DMSO. Product release from substrate hydrolysis (75 mM) was monitored continuously over a period of 10 min.

Inhibition assay for the Tryptic Activity of the 20S Proteasome

Human 20S proteasome was incubated at 30 °C at a final concentration of 0.0025 mg·mL^{-1} with the test compound present at 10 μM (screening assay) or at variable concentrations (continuous assay). The reaction buffer consisted of 50 mM Tris buffer pH 7.4, 50 mM NaCl, 0.5 mM EDTA, 0.03% SDS and 7.5% DMSO. Product release from substrate hydrolysis (85 mM) was monitored continuously over a period of 10 min.

Inhibition assay for the Post-Glutamyl Peptide Hydrolyzing Activity of the 20S Proteasome

Human 20S proteasome was incubated at 30 °C at a final concentration of 0.004 mg·mL^{-1} with the test compound present at 10 μM (screening assay) or variable concentrations. The reaction buffer consisted of 50 mM Tris buffer pH 7.5 containing 25 mM KCl, 10 mM NaCl, 1 mM $MgCl_2$, 0.03% SDS, 5% DMSO. Product release from substrate hydrolysis (80 mM) was monitored continuously over a period of 10 min.

Assay for Bovine Pancreatic α-Chymotrypsin Inhibition

The enzyme (250 mg·mL^{-1}) was incubated at 20 °C with the test compound present at 10 μM (screening assay). The reaction buffer consisted of 50 mM Tris buffer pH 8.0 containing 100 mM NaCl, 5 mM EDTA and 7.5% DMSO. Product release from substrate hydrolysis (75 mM final concentration, Suc-Leu-Leu-Val-Tyr-AMC from Bachem) was determined over a period of 10 min.

Assay for Cathepsin-B and Cathepsin-L Inhibition

Assays were performed at 25 °C in a 20 mM Tris-HCl buffer, pH 6.0, containing 5 mM EDTA, 2.5 mM DTT, 200 mM NaCl, 0.005% Brij 35 in a total volume of 285 μL. Substrate (Cbz-Phe-Arg-AMC for both enzymes) and inhibitor stock solutions were prepared in DMSO (10% final concentration) and were diluted with assay buffer; enzymes were held in buffer. The final substrate concentration for inhibition assays was between 10.0 and 81.0 mM; the final enzyme concentration was 53 ng mL^{-1} for CL (*P. tetraurelia*) and 58 pg mL^{-1} for CB (recombinant, human liver). Inhibitors were used at concentrations between 0.35 μM and 140 μM. Product release from substrate hydrolysis was determined over a period of 10 min.
